# Morpholine-4-carboxamidinium ethyl carbonate

**DOI:** 10.1107/S1600536812046922

**Published:** 2012-11-24

**Authors:** Ioannis Tiritiris

**Affiliations:** a Fakultät Chemie/Organische Chemie, Hochschule Aalen, Beethovenstrasse 1, D-73430 Aalen, Germany

## Abstract

The asymmetric unit of the title salt, C_5_H_12_N_3_O^+^·C_3_H_5_O_3_^−^, contains two carboxamidinium and two ethyl carbonate ions. In the crystal, the C—N bond lengths in the central CN_3_ units of the cations range between 1.324 (2) and 1.352 (2) Å, indicating partial double-bond character. The central C atoms are bonded to the three N atoms in a nearly ideal trigonal–planar geometry and the positive charges are delocalized in the CN_3_ planes. The morpholine rings are in chair conformations. The C—O bond lengths in both ethyl carbonate ions are characteristic for delocalized double bonds [1.243 (2)–1.251 (2) Å] and typical single bonds [1.368 (2) and 1.375 (2) Å]. In the crystal, N—H⋯O hydrogen bonds between cations and anions generate a two-dimensional network in the *ac* plane.

## Related literature

For the synthesis and crystal structures of guanidinium hydrogen carbonates, see: Tiritiris *et al.* (2011 ▶). For the crystal structure of 4-morpholine­carboxamidine, see: Tiritiris (2012*a* ▶). For the crystal structure of piperidine-1-carboxamidinium ethyl carbonate, see: Tiritiris (2012*b* ▶).
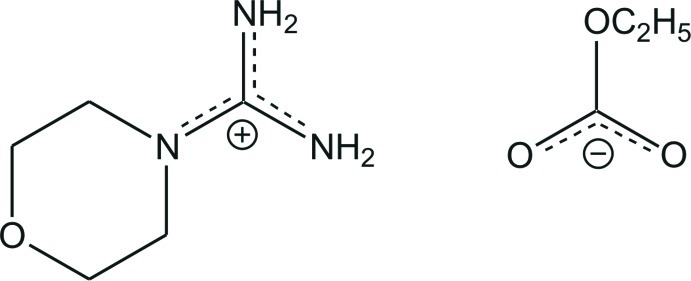


## Experimental

### 

#### Crystal data

C_5_H_12_N_3_O^+^·C_3_H_5_O_3_^−^
*M* *_r_* = 219.25
Monoclinic, 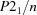

*a* = 10.2163 (5) Å
*b* = 20.8874 (9) Å
*c* = 10.4616 (5) Å
β = 109.505 (2)°
*V* = 2104.31 (17) Å^3^
*Z* = 8
Mo *K*α radiation
μ = 0.11 mm^−1^
*T* = 100 K
0.30 × 0.25 × 0.15 mm

#### Data collection

Bruker–Nonius KappaCCD diffractometer
9902 measured reflections
5199 independent reflections
2981 reflections with *I* > 2σ(*I*)
*R*_int_ = 0.055

#### Refinement

*R*[*F* ^2^ > 2σ(*F* ^2^)] = 0.050
*wR*(*F* ^2^) = 0.112
*S* = 1.00
5199 reflections
305 parameters
H atoms treated by a mixture of independent and constrained refinement
Δρ_max_ = 0.25 e Å^−3^
Δρ_min_ = −0.30 e Å^−3^

### 

Data collection: *COLLECT* (Hooft, 2004 ▶); cell refinement: *SCALEPACK* (Otwinowski & Minor, 1997 ▶); data reduction: *SCALEPACK*; program(s) used to solve structure: *SHELXS97* (Sheldrick, 2008 ▶); program(s) used to refine structure: *SHELXL97* (Sheldrick, 2008 ▶); molecular graphics: *DIAMOND* (Brandenburg & Putz, 2005 ▶); software used to prepare material for publication: *SHELXL97*.

## Supplementary Material

Crystal structure: contains datablock(s) I, global. DOI: 10.1107/S1600536812046922/kp2441sup1.cif

Structure factors: contains datablock(s) I. DOI: 10.1107/S1600536812046922/kp2441Isup2.hkl

Additional supplementary materials:  crystallographic information; 3D view; checkCIF report

## Figures and Tables

**Table 1:** Hydrogen-bond geometry (Å, °)

*D*—H⋯*A*	*D*—H	H⋯*A*	*D*⋯*A*	*D*—H⋯*A*
N1—H11⋯O4^i^	0.84 (2)	2.12 (2)	2.944 (1)	168 (1)
N1—H12⋯O3^ii^	0.89 (2)	1.91 (2)	2.795 (1)	174 (1)
N2—H21⋯O6	0.85 (2)	1.97 (2)	2.807 (1)	168 (1)
N2—H22⋯O4^ii^	0.92 (2)	1.95 (2)	2.851 (1)	164 (1)
N4—H41⋯O6^ii^	0.86 (2)	1.97 (2)	2.817 (1)	167 (1)
N4—H42⋯O7^i^	0.93 (2)	2.00 (2)	2.889 (1)	159 (1)
N5—H51⋯O7^ii^	0.90 (2)	1.99 (2)	2.879 (1)	172 (1)
N5—H52⋯O3^iii^	0.90 (2)	1.94 (2)	2.776 (1)	154 (1)

Symmetry codes: (i) 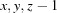; (ii) 
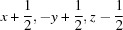
; (iii) 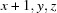.

**Fig. 1.**
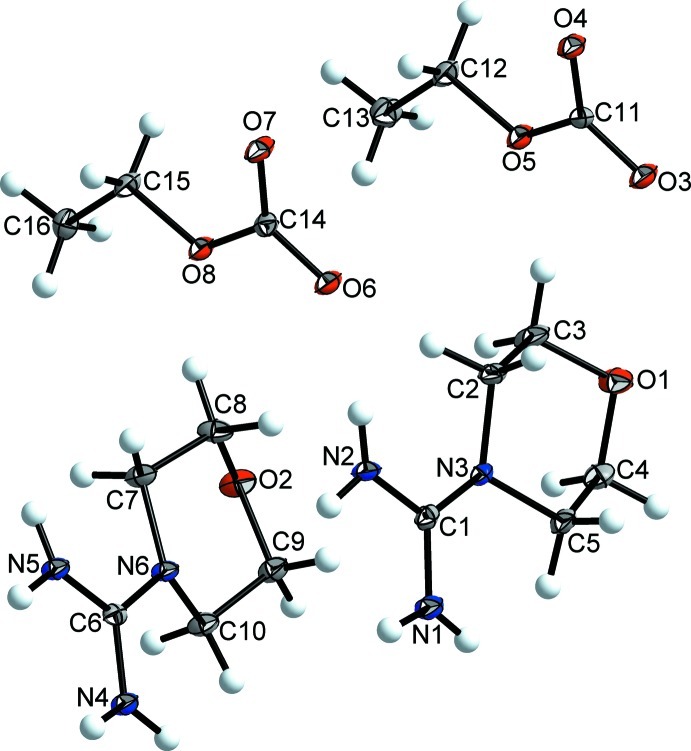
The structure of the title compound with displacement ellipsoids at the 50% probability level.

**Fig. 2.**
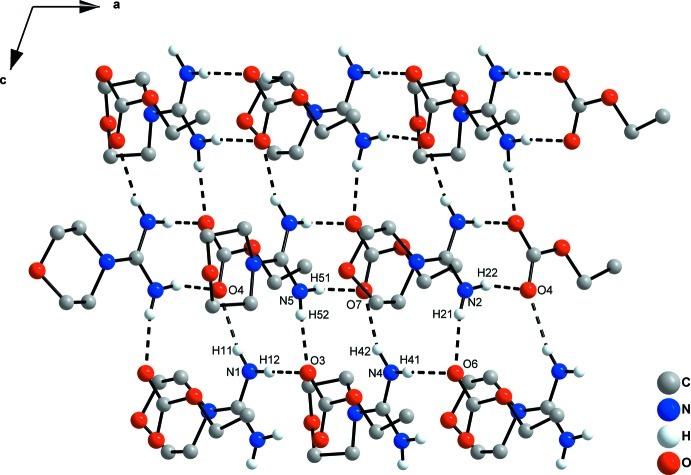
N–H···O hydrogen bonds generating a two-dimensional network in the (ac) plane. The hydrogen bonds are indicated by dashed lines.

## supplementary crystallographic information

### Comment

The reaction of several guanidines with CO_2_ in undried aprotic solvents are well described in the literature (Tiritiris *et al.*, 2011). Here, the corresponding guanidinium hydrogen carbonate salts were obtained and their crystal structures could be determined. By reacting carboxamidines with CO_2_ we first used aprotic solvents and due to their water content, sparingly soluble and non crystalline hydrogen carbonate salts were also formed. By using alcohols as solvents for the reaction, we obtained a few crystalline alkyl carbonate salts. One of them is the here presented title compound. According to the structure analysis, the asymmetric unit contains two carboxamidinium and two ethyl carbonate ions. The C–N bonds of the CN_3_ units are ranging from 1.324 (2) to 1.352 (2) Å, showing partial double-bond character. The N–C1–N and N–C6–N angles are indicating a nearly ideal trigonal-planar surrounding of the carbon centres by the nitrogen atoms. The positive charges are completely delocalized on the CN_3_ planes (Fig. 1). The structural parameters of the morpholine rings in the here presented title compound agree very well with the data obtained from the X-ray analysis of the starting compound 4-morpholinecarboxamidine (Tiritiris, 2012*a*). The morpholine rings adopt a chair conformation. The C–O bond lengths in both ethyl carbonate ions indicate evenly distributed double bonds [1.243 (2)–1.251 (2) Å] and typical single bonds [1.368 (2) and 1.375 (2) Å]. The data fit with the C–O bond lengths and angles of the anion in piperidine-1-carboxamidinium ethyl carbonate (Tiritiris, 2012*b*). In the crystal structure, strong N—H···O hydrogen bonds between hydrogen atoms of carboxamidinium ions and oxygen atoms of neighboring ethyl carbonate ions are observed, generating an infinite two-dimensional network [*d*(H···O) = 1.91 (2)–2.12 (2) Å] (Tab. 1) with base vectors [0 0 1] and [1 0 0] (Fig. 2). In contrast to the crystal structure of 4-morpholinecarboxamidine (Tiritiris, 2012*a*), the oxygen atoms of the morpholine rings are not involved in the N—H···O hydrogen bonding system.

### Experimental

The title compound was prepared by bubbling excess CO_2_ gas into an ethanolic solution of 2.0 g (15.5 mmol) 4-morpholinecarboxamidine (Tiritiris, 2012*a*). The resulting colorless precipitate was recrystallized from a small amount of ethanol and single crystals suitable for X-ray analysis were obtained. Yield: 3.05 g (90%). ^1^H NMR (500 MHz, D_2_O/DSS): δ = 1.17–1.20 [t, 3 H, –CH_3_], 3.49–3.52 [m, 4 H, –CH_2_], 3.64–3.68 [q, 2 H, –CH_2_], 3.80–3.83 [m, 4 H, –CH_2_]. Because of the H/D exchange, the hydrogen atoms of the –NH_2_ groups were not observed. ^13^C NMR (125 MHz, D_2_O/DSS): δ = 16.8 (–CH_3_), 45.2 (–CH_2_), 57.4 (–CH_2_), 65.4 (–CH_2_), 156.6 (N_3_C^+^), 160.3 (C═O).

### Refinement

The N-bound H atoms were located in a difference Fourier map and were refined freely [N—H = 0.84 (2)–0.93 (2) Å]. The hydrogen atoms of the methyl groups were allowed to rotate with a fixed angle around the C–C bond to best fit the experimental electron density, with *U*(H) set to 1.5 *U*_eq_(C) and d(C—H) = 0.98 Å. The H atoms of the methylene groups were placed in calculated positions with d(C—H) = 0.99 Å. They were included in the refinement in the riding model approximation, with *U*(H) set to 1.2 *U*_eq_(C).

### Crystal data

**Table:**

C_5_H_12_N_3_O^+^·C_3_H_5_O_3_^−^	*F*(000) = 944
*M**_r_* = 219.25	*D*_x_ = 1.384 Mg m^−^^3^
Monoclinic, *P*2_1_/*n*	Melting point: 413 K
Hall symbol: -P 2yn	Mo *K*α radiation, λ = 0.71073 Å
*a* = 10.2163 (5) Å	Cell parameters from 5142 reflections
*b* = 20.8874 (9) Å	θ = 0.4–28.3°
*c* = 10.4616 (5) Å	µ = 0.11 mm^−^^1^
β = 109.505 (2)°	*T* = 100 K
*V* = 2104.31 (17) Å^3^	Block, colourless
*Z* = 8	0.30 × 0.25 × 0.15 mm

### Data collection

**Table:**

Bruker–Nonius KappaCCD diffractometer	2981 reflections with *I* > 2σ(*I*)
Radiation source: sealed tube	*R*_int_ = 0.055
Graphite monochromator	θ_max_ = 28.3°, θ_min_ = 2.3°
φ scans, and ω scans	*h* = −13→13
9902 measured reflections	*k* = −27→27
5199 independent reflections	*l* = −13→13

### Refinement

**Table:**

Refinement on *F*^2^	Primary atom site location: structure-invariant direct methods
Least-squares matrix: full	Secondary atom site location: difference Fourier map
*R*[*F*^2^ > 2σ(*F*^2^)] = 0.050	Hydrogen site location: difference Fourier map
*wR*(*F*^2^) = 0.112	H atoms treated by a mixture of independent and constrained refinement
*S* = 1.00	*w* = 1/[σ^2^(*F*_o_^2^) + (0.0468*P*)^2^] where *P* = (*F*_o_^2^ + 2*F*_c_^2^)/3
5199 reflections	(Δ/σ)_max_ < 0.001
305 parameters	Δρ_max_ = 0.25 e Å^−^^3^
0 restraints	Δρ_min_ = −0.30 e Å^−^^3^

### Special details

**Table:**

Geometry. All e.s.d.'s (except the e.s.d. in the dihedral angle between two l.s. planes) are estimated using the full covariance matrix. The cell e.s.d.'s are taken into account individually in the estimation of e.s.d.'s in distances, angles and torsion angles; correlations between e.s.d.'s in cell parameters are only used when they are defined by crystal symmetry. An approximate (isotropic) treatment of cell e.s.d.'s is used for estimating e.s.d.'s involving l.s. planes.
Refinement. Refinement of *F*^2^ against ALL reflections. The weighted *R*-factor *wR* and goodness of fit *S* are based on *F*^2^, conventional *R*-factors *R* are based on *F*, with *F* set to zero for negative *F*^2^. The threshold expression of *F*^2^ > σ(*F*^2^) is used only for calculating *R*-factors(gt) *etc*. and is not relevant to the choice of reflections for refinement. *R*-factors based on *F*^2^ are statistically about twice as large as those based on *F*, and *R*- factors based on ALL data will be even larger.

### Fractional atomic coordinates and isotropic or equivalent isotropic displacement parameters (Å^2^)

**Table:**

	*x*	*y*	*z*	*U*_iso_*/*U*_eq_	
C1	0.14782 (18)	0.19683 (9)	0.08078 (17)	0.0138 (4)	
N1	0.14407 (18)	0.21777 (9)	−0.03987 (16)	0.0186 (4)	
H11	0.082 (2)	0.2060 (11)	−0.111 (2)	0.027 (6)*	
H12	0.209 (2)	0.2468 (11)	−0.039 (2)	0.028 (6)*	
N2	0.24717 (16)	0.21987 (9)	0.18879 (16)	0.0168 (4)	
H21	0.2567 (18)	0.2083 (9)	0.269 (2)	0.013 (5)*	
H22	0.308 (2)	0.2486 (11)	0.171 (2)	0.032 (6)*	
N3	0.05782 (14)	0.15198 (8)	0.09382 (13)	0.0138 (3)	
C2	0.04415 (18)	0.14053 (10)	0.22786 (16)	0.0174 (4)	
H2A	−0.0106	0.1755	0.2491	0.021*	
H2B	0.1374	0.1404	0.2982	0.021*	
C3	−0.02671 (19)	0.07728 (10)	0.22976 (17)	0.0239 (5)	
H3A	0.0343	0.0421	0.2203	0.029*	
H3B	−0.0410	0.0721	0.3182	0.029*	
O1	−0.15763 (13)	0.07261 (7)	0.12343 (12)	0.0227 (3)	
C4	−0.13597 (19)	0.07748 (10)	−0.00376 (17)	0.0194 (4)	
H4A	−0.2261	0.0727	−0.0777	0.023*	
H4B	−0.0746	0.0422	−0.0120	0.023*	
C5	−0.07145 (18)	0.14066 (9)	−0.01951 (17)	0.0168 (4)	
H5A	−0.0515	0.1408	−0.1058	0.020*	
H5B	−0.1379	0.1757	−0.0233	0.020*	
C6	0.61678 (17)	0.19115 (9)	0.08546 (16)	0.0122 (4)	
N4	0.60204 (17)	0.21566 (9)	−0.03589 (15)	0.0161 (4)	
H41	0.656 (2)	0.2472 (11)	−0.038 (2)	0.026 (6)*	
H42	0.533 (2)	0.2013 (12)	−0.113 (2)	0.041 (7)*	
N5	0.71439 (16)	0.21538 (8)	0.19268 (15)	0.0144 (4)	
H51	0.773 (2)	0.2459 (11)	0.185 (2)	0.023 (6)*	
H52	0.745 (2)	0.1933 (11)	0.271 (2)	0.035 (6)*	
N6	0.53305 (14)	0.14360 (7)	0.10007 (13)	0.0134 (3)	
C7	0.56842 (19)	0.11205 (10)	0.23299 (16)	0.0176 (4)	
H7A	0.5906	0.1449	0.3053	0.021*	
H7B	0.6517	0.0850	0.2480	0.021*	
C8	0.4493 (2)	0.07110 (10)	0.24096 (17)	0.0189 (4)	
H8A	0.4791	0.0472	0.3277	0.023*	
H8B	0.3709	0.0991	0.2401	0.023*	
O2	0.40359 (13)	0.02701 (6)	0.13163 (11)	0.0198 (3)	
C9	0.35206 (18)	0.06230 (10)	0.00799 (17)	0.0182 (4)	
H9A	0.2758	0.0907	0.0117	0.022*	
H9B	0.3137	0.0321	−0.0684	0.022*	
C10	0.46389 (18)	0.10203 (9)	−0.01694 (17)	0.0169 (4)	
H10A	0.5335	0.0734	−0.0341	0.020*	
H10B	0.4226	0.1288	−0.0986	0.020*	
C11	−0.07231 (18)	0.16716 (9)	0.57058 (16)	0.0133 (4)	
O3	−0.16622 (12)	0.18499 (7)	0.46539 (11)	0.0196 (3)	
O4	−0.04966 (12)	0.18664 (6)	0.68830 (11)	0.0179 (3)	
O5	0.01060 (12)	0.11991 (7)	0.54704 (11)	0.0181 (3)	
C12	0.12224 (18)	0.09664 (10)	0.66226 (17)	0.0188 (4)	
H12A	0.0849	0.0761	0.7281	0.023*	
H12B	0.1836	0.1324	0.7081	0.023*	
C13	0.20159 (19)	0.04887 (10)	0.60972 (18)	0.0224 (5)	
H13A	0.2355	0.0695	0.5427	0.034*	
H13B	0.1405	0.0131	0.5671	0.034*	
H13C	0.2806	0.0328	0.6851	0.034*	
C14	0.40406 (17)	0.17608 (9)	0.57044 (16)	0.0130 (4)	
O6	0.31041 (12)	0.19554 (6)	0.46676 (11)	0.0180 (3)	
O7	0.42103 (13)	0.19133 (6)	0.69029 (11)	0.0183 (3)	
O8	0.49287 (12)	0.13310 (6)	0.54411 (11)	0.0166 (3)	
C15	0.60379 (18)	0.10913 (10)	0.65905 (17)	0.0179 (4)	
H15A	0.5655	0.0877	0.7232	0.021*	
H15B	0.6641	0.1448	0.7070	0.021*	
C16	0.68612 (19)	0.06219 (10)	0.60715 (18)	0.0199 (4)	
H16A	0.6270	0.0257	0.5651	0.030*	
H16B	0.7659	0.0470	0.6828	0.030*	
H16C	0.7190	0.0833	0.5398	0.030*	

### Atomic displacement parameters (Å^2^)

**Table:**

	*U*^11^	*U*^22^	*U*^33^	*U*^12^	*U*^13^	*U*^23^
C1	0.0137 (9)	0.0140 (11)	0.0127 (9)	0.0045 (8)	0.0031 (7)	0.0005 (7)
N1	0.0180 (9)	0.0236 (10)	0.0114 (8)	−0.0068 (8)	0.0011 (7)	0.0008 (7)
N2	0.0189 (9)	0.0223 (10)	0.0080 (8)	−0.0050 (7)	0.0031 (7)	0.0016 (7)
N3	0.0118 (7)	0.0192 (9)	0.0088 (7)	−0.0017 (7)	0.0012 (6)	−0.0003 (6)
C2	0.0178 (9)	0.0251 (12)	0.0085 (8)	−0.0042 (8)	0.0033 (7)	−0.0007 (8)
C3	0.0261 (11)	0.0301 (13)	0.0102 (9)	−0.0091 (9)	−0.0011 (8)	0.0013 (8)
O1	0.0251 (7)	0.0279 (9)	0.0138 (6)	−0.0121 (6)	0.0048 (5)	−0.0029 (6)
C4	0.0217 (10)	0.0216 (12)	0.0130 (9)	−0.0007 (9)	0.0032 (8)	−0.0024 (8)
C5	0.0148 (9)	0.0201 (11)	0.0115 (8)	−0.0027 (8)	−0.0007 (7)	0.0001 (8)
C6	0.0143 (9)	0.0123 (10)	0.0107 (9)	0.0040 (8)	0.0051 (7)	−0.0004 (7)
N4	0.0166 (8)	0.0182 (10)	0.0107 (8)	−0.0043 (8)	0.0009 (7)	0.0016 (7)
N5	0.0164 (8)	0.0145 (10)	0.0103 (8)	−0.0029 (7)	0.0018 (6)	0.0000 (6)
N6	0.0158 (8)	0.0148 (9)	0.0083 (7)	−0.0017 (7)	0.0023 (6)	−0.0007 (6)
C7	0.0218 (10)	0.0185 (11)	0.0105 (9)	−0.0053 (8)	0.0027 (7)	0.0016 (7)
C8	0.0268 (10)	0.0179 (12)	0.0125 (9)	−0.0045 (9)	0.0074 (8)	−0.0022 (8)
O2	0.0281 (7)	0.0145 (8)	0.0147 (6)	−0.0057 (6)	0.0043 (5)	−0.0011 (5)
C9	0.0187 (10)	0.0187 (12)	0.0137 (9)	−0.0021 (8)	0.0006 (7)	0.0008 (8)
C10	0.0200 (10)	0.0167 (11)	0.0117 (8)	−0.0022 (8)	0.0025 (7)	−0.0013 (7)
C11	0.0130 (9)	0.0173 (11)	0.0095 (9)	−0.0009 (8)	0.0037 (7)	0.0013 (7)
O3	0.0191 (7)	0.0258 (9)	0.0101 (6)	0.0043 (6)	−0.0001 (5)	0.0003 (5)
O4	0.0183 (7)	0.0231 (8)	0.0102 (6)	0.0030 (6)	0.0020 (5)	−0.0016 (5)
O5	0.0175 (7)	0.0238 (8)	0.0108 (6)	0.0056 (6)	0.0016 (5)	−0.0002 (5)
C12	0.0167 (10)	0.0226 (12)	0.0138 (9)	0.0045 (8)	0.0007 (7)	0.0022 (8)
C13	0.0234 (10)	0.0219 (12)	0.0211 (10)	0.0027 (9)	0.0064 (8)	0.0014 (8)
C14	0.0131 (9)	0.0131 (11)	0.0116 (9)	−0.0027 (8)	0.0027 (7)	0.0001 (7)
O6	0.0179 (7)	0.0231 (8)	0.0101 (6)	0.0050 (6)	0.0010 (5)	0.0010 (5)
O7	0.0208 (7)	0.0215 (8)	0.0096 (6)	0.0050 (6)	0.0010 (5)	−0.0021 (5)
O8	0.0163 (7)	0.0211 (8)	0.0103 (6)	0.0056 (6)	0.0014 (5)	0.0005 (5)
C15	0.0177 (9)	0.0214 (12)	0.0113 (9)	0.0057 (8)	0.0005 (7)	0.0012 (8)
C16	0.0171 (10)	0.0218 (12)	0.0198 (10)	0.0037 (8)	0.0047 (8)	0.0023 (8)

### Geometric parameters (Å, º)

**Table:**

C1—N1	1.324 (2)	C7—H7A	0.9900
C1—N2	1.331 (2)	C7—H7B	0.9900
C1—N3	1.351 (2)	C8—O2	1.420 (2)
N1—H11	0.84 (2)	C8—H8A	0.9900
N1—H12	0.89 (2)	C8—H8B	0.9900
N2—H21	0.85 (2)	O2—C9	1.428 (2)
N2—H22	0.92 (2)	C9—C10	1.504 (3)
N3—C5	1.470 (2)	C9—H9A	0.9900
N3—C2	1.474 (2)	C9—H9B	0.9900
C2—C3	1.510 (3)	C10—H10A	0.9900
C2—H2A	0.9900	C10—H10B	0.9900
C2—H2B	0.9900	C11—O4	1.243 (2)
C3—O1	1.429 (2)	C11—O3	1.251 (2)
C3—H3A	0.9900	C11—O5	1.375 (2)
C3—H3B	0.9900	O5—C12	1.439 (2)
O1—C4	1.424 (2)	C12—C13	1.501 (3)
C4—C5	1.508 (3)	C12—H12A	0.9900
C4—H4A	0.9900	C12—H12B	0.9900
C4—H4B	0.9900	C13—H13A	0.9800
C5—H5A	0.9900	C13—H13B	0.9800
C5—H5B	0.9900	C13—H13C	0.9800
C6—N5	1.327 (2)	C14—O7	1.248 (2)
C6—N4	1.330 (2)	C14—O6	1.2507 (19)
C6—N6	1.352 (2)	C14—O8	1.368 (2)
N4—H41	0.86 (2)	O8—C15	1.4390 (19)
N4—H42	0.93 (2)	C15—C16	1.506 (3)
N5—H51	0.90 (2)	C15—H15A	0.9900
N5—H52	0.90 (2)	C15—H15B	0.9900
N6—C7	1.471 (2)	C16—H16A	0.9800
N6—C10	1.474 (2)	C16—H16B	0.9800
C7—C8	1.512 (3)	C16—H16C	0.9800
			
N1—C1—N2	117.44 (18)	C8—C7—H7B	109.5
N1—C1—N3	121.41 (16)	H7A—C7—H7B	108.0
N2—C1—N3	121.10 (16)	O2—C8—C7	112.11 (14)
C1—N1—H11	121.6 (14)	O2—C8—H8A	109.2
C1—N1—H12	115.2 (13)	C7—C8—H8A	109.2
H11—N1—H12	123.1 (19)	O2—C8—H8B	109.2
C1—N2—H21	122.7 (13)	C7—C8—H8B	109.2
C1—N2—H22	116.0 (13)	H8A—C8—H8B	107.9
H21—N2—H22	121.3 (17)	C8—O2—C9	108.51 (14)
C1—N3—C5	119.25 (14)	O2—C9—C10	111.74 (14)
C1—N3—C2	119.50 (14)	O2—C9—H9A	109.3
C5—N3—C2	113.33 (13)	C10—C9—H9A	109.3
N3—C2—C3	110.65 (14)	O2—C9—H9B	109.3
N3—C2—H2A	109.5	C10—C9—H9B	109.3
C3—C2—H2A	109.5	H9A—C9—H9B	107.9
N3—C2—H2B	109.5	N6—C10—C9	111.24 (14)
C3—C2—H2B	109.5	N6—C10—H10A	109.4
H2A—C2—H2B	108.1	C9—C10—H10A	109.4
O1—C3—C2	112.19 (15)	N6—C10—H10B	109.4
O1—C3—H3A	109.2	C9—C10—H10B	109.4
C2—C3—H3A	109.2	H10A—C10—H10B	108.0
O1—C3—H3B	109.2	O4—C11—O3	127.48 (17)
C2—C3—H3B	109.2	O4—C11—O5	119.35 (15)
H3A—C3—H3B	107.9	O3—C11—O5	113.17 (14)
C4—O1—C3	109.00 (13)	C11—O5—C12	117.10 (13)
O1—C4—C5	112.00 (15)	O5—C12—C13	106.96 (14)
O1—C4—H4A	109.2	O5—C12—H12A	110.3
C5—C4—H4A	109.2	C13—C12—H12A	110.3
O1—C4—H4B	109.2	O5—C12—H12B	110.3
C5—C4—H4B	109.2	C13—C12—H12B	110.3
H4A—C4—H4B	107.9	H12A—C12—H12B	108.6
N3—C5—C4	111.13 (14)	C12—C13—H13A	109.5
N3—C5—H5A	109.4	C12—C13—H13B	109.5
C4—C5—H5A	109.4	H13A—C13—H13B	109.5
N3—C5—H5B	109.4	C12—C13—H13C	109.5
C4—C5—H5B	109.4	H13A—C13—H13C	109.5
H5A—C5—H5B	108.0	H13B—C13—H13C	109.5
N5—C6—N4	118.27 (17)	O7—C14—O6	126.70 (17)
N5—C6—N6	120.64 (15)	O7—C14—O8	119.38 (14)
N4—C6—N6	121.08 (16)	O6—C14—O8	113.91 (14)
C6—N4—H41	116.3 (13)	C14—O8—C15	116.75 (12)
C6—N4—H42	121.0 (14)	O8—C15—C16	107.70 (14)
H41—N4—H42	122.5 (19)	O8—C15—H15A	110.2
C6—N5—H51	121.8 (13)	C16—C15—H15A	110.2
C6—N5—H52	120.7 (14)	O8—C15—H15B	110.2
H51—N5—H52	114.0 (18)	C16—C15—H15B	110.2
C6—N6—C7	118.09 (13)	H15A—C15—H15B	108.5
C6—N6—C10	118.98 (14)	C15—C16—H16A	109.5
C7—N6—C10	114.80 (15)	C15—C16—H16B	109.5
N6—C7—C8	110.93 (14)	H16A—C16—H16B	109.5
N6—C7—H7A	109.5	C15—C16—H16C	109.5
C8—C7—H7A	109.5	H16A—C16—H16C	109.5
N6—C7—H7B	109.5	H16B—C16—H16C	109.5
			
N1—C1—N3—C5	−19.3 (3)	N4—C6—N6—C10	23.7 (2)
N2—C1—N3—C5	163.26 (16)	C6—N6—C7—C8	167.46 (16)
N1—C1—N3—C2	−166.30 (17)	C10—N6—C7—C8	−44.1 (2)
N2—C1—N3—C2	16.3 (3)	N6—C7—C8—O2	53.4 (2)
C1—N3—C2—C3	−163.24 (16)	C7—C8—O2—C9	−62.83 (19)
C5—N3—C2—C3	47.9 (2)	C8—O2—C9—C10	62.98 (19)
N3—C2—C3—O1	−54.7 (2)	C6—N6—C10—C9	−167.27 (15)
C2—C3—O1—C4	61.1 (2)	C7—N6—C10—C9	44.6 (2)
C3—O1—C4—C5	−60.9 (2)	O2—C9—C10—N6	−53.8 (2)
C1—N3—C5—C4	163.03 (16)	O4—C11—O5—C12	−1.3 (2)
C2—N3—C5—C4	−48.1 (2)	O3—C11—O5—C12	179.62 (15)
O1—C4—C5—N3	54.7 (2)	C11—O5—C12—C13	−176.69 (15)
N5—C6—N6—C7	−10.5 (2)	O7—C14—O8—C15	−1.3 (2)
N4—C6—N6—C7	170.74 (16)	O6—C14—O8—C15	179.56 (15)
N5—C6—N6—C10	−157.57 (16)	C14—O8—C15—C16	179.01 (15)

### Hydrogen-bond geometry (Å, º)

**Table:**

*D*—H···*A*	*D*—H	H···*A*	*D*···*A*	*D*—H···*A*
N1—H11···O4^i^	0.84 (2)	2.12 (2)	2.944 (1)	168 (1)
N1—H12···O3^ii^	0.89 (2)	1.91 (2)	2.795 (1)	174 (1)
N2—H21···O6	0.85 (2)	1.97 (2)	2.807 (1)	168 (1)
N2—H22···O4^ii^	0.92 (2)	1.95 (2)	2.851 (1)	164 (1)
N4—H41···O6^ii^	0.86 (2)	1.97 (2)	2.817 (1)	167 (1)
N4—H42···O7^i^	0.93 (2)	2.00 (2)	2.889 (1)	159 (1)
N5—H51···O7^ii^	0.90 (2)	1.99 (2)	2.879 (1)	172 (1)
N5—H52···O3^iii^	0.90 (2)	1.94 (2)	2.776 (1)	154 (1)

Symmetry codes: (i) *x*, *y*, *z*−1; (ii) *x*+1/2, −*y*+1/2, *z*−1/2; (iii) *x*+1, *y*, *z*.
